# Attitudes and concerns of undergraduate university health sciences students in Croatia regarding complete switch to e-learning during COVID-19 pandemic: a survey

**DOI:** 10.1186/s12909-020-02343-7

**Published:** 2020-11-10

**Authors:** Livia Puljak, Marta Čivljak, Ana Haramina, Snježana Mališa, Dalibor Čavić, Dinko Klinec, Diana Aranza, Jasna Mesarić, Nataša Skitarelić, Sanja Zoranić, Dijana Majstorović, Marijana Neuberg, Štefica Mikšić, Kata Ivanišević

**Affiliations:** 1 Center for Evidence-Based Medicine and Health Care, Catholic University of Croatia, Ilica 242, 10000 Zagreb, Croatia; 2 University Department of Health Studies, University of Split, Split, Croatia; 3 Libertas International University, Zagreb, Croatia; 4 Department of Health Studies, University of Zadar, Zadar, Croatia; 5 Department of Nursing, University of Dubrovnik, Dubrovnik, Croatia; 6 Medical school, Juraj Dobrila University of Pula, Pula, Croatia; 7 Department of Nursing, University North, Varaždin, Croatia; 8 Faculty of Dental Medicine and Health Osijek, Osijek, Croatia; 9 Faculty of Health Studies, University of Rijeka, Rijeka, Croatia

**Keywords:** SARS-COV-2, COVID-19, Coronavirus, Pandemic, Croatia, Education, Medical education, Health sciences

## Abstract

**Background:**

Croatia has closed all educational institutions after 32 cases of SARS-CoV-2 infection were confirmed and switched to exclusive e-learning. Health sciences university students may have been particularly affected with this change due to a lack of practical education. It is not known how health sciences students and schools have adjusted to exclusive e-learning. This study aimed to explore attitudes and concerns of health sciences students in Croatia regarding the complete switch to e-learning during the COVID-19 pandemic.

**Methods:**

Eligible participants were students from 9 institutions offering university-level health sciences education in Croatia enrolled in the academic year 2019/2010, and participating in e-learning. Data were collected with a questionnaire distributed via email during April/May 2020.

**Results:**

A total of 2520 students (aged 25.7 ± 7.7 years) responded to the questionnaire (70.3% response rate). General satisfaction with exclusive e-learning was rated with average grade of 3.7 out of 5. Compared with previous education, exclusive e-learning was rated with average grade of 3.2 out of 5. Compared to classroom learning, equal or higher motivation to attend exclusive e-learning was reported by 64.4% of participants. With a longer duration of exclusive e-learning, equal or higher motivation was reported by 65.5% of participants. Less than half of the students indicated they felt deprived or concerned due to the lack of practical lessons. Most participants indicated that in the future, they would prefer to combine classic classroom and e-learning (*N* = 1403; 55.7%).

**Conclusions:**

Most health sciences students were satisfied with the exclusive e-learning, as well as their personal and institutional adjustment to it. Students’ feedback can help institutions to improve the exclusive e-learning experience for students in the time of the pandemic.

**Supplementary Information:**

The online version contains supplementary material available at 10.1186/s12909-020-02343-7.

## Background

The first case of SARS-CoV-2 infection in Croatia was confirmed on February 25, 2020; this was the case of a 25-year-old man who returned from Milan, Italy on February 20, 2020 [1]. On March 13, 2020, when Croatia had 32 confirmed cases of SARS-CoV-2 infection [2], the Croatian government decided to close all educational institutions in Croatia – the decision coming into effect on March 16, 2020.

On April 9, 2020 Croatia had 1343 confirmed cases of SARS-CoV-2 infection and 19 deceased due to COVID-19. Epidemiological growth curve remained linear even though Croatia’s capital Zagreb suffered from an earthquake with a magnitude of 5.5 Richter on March 22, 2020, causing considerable material damage, an exodus of people on streets and concerns that this will further fuel the epidemic [2].

The Croatian government decided that all school and university lessons need to be delivered via distance learning [3, 4]. The initial decision of the Croatian government was to suspend in-person learning in Croatian educational institutions for 2 weeks, but the closure was extended.

University education was, thus, organized via distance learning, but medical education has been disrupted in a particular way. In medical and health sciences university programs theoretical lessons only can take part via distance learning, since teaching hospitals and clinics have suspended practical parts of students’ education, as a measure of curbing the epidemic in health institutions. Organizing and studying practical medical education is particularly challenging in times of pandemic [5]. However, if we neglect what is happening with health professional education in challenging times, we may jeopardize the professional development of students and future care of patients.

In the literature, we were unable to find large-scale studies on the national level about adjustment of health sciences students and institutions to the COVID-19 pandemic. A study of medical students in Singapore, regarding teaching anatomy over Zoom, described the general satisfaction of students [6]. A qualitative study of 32 nursing students in Spain who switched from face-to-face to e-learning during the pandemic indicated that e-learning was most troubling for older students, those from rural areas, with work and family responsibilities and students with limited electronic resources [7]. Another qualitative study of 60 medical students from Saudi Arabia showed that online learning was well-received and that students recognized some advantages of such education, such as time-saving and improved utility of time. However, students indicated that they encountered multiple challenges as well, including methodological issues, problems with content perception, technical and behavioral issues during educational sessions, and online exams. The majority of the preclinical students included in the study indicated that they would prefer e-learning during the next academic year as well [8].

Taking into account the assumptions of student-centered education [9–12], which, among other things, emphasizes the importance of the quality of personal learning experience and the circumstances of the COVID-19 pandemic, which are incomparable with previous experiences, it is justified to start scientific research by describing and determining new educational conditions for health sciences students.

Thus, this study aimed to explore attitudes and concerns of health sciences students in Croatia regarding the complete switch to e-learning during the COVID-19 pandemic.

## Methods

### Study design

This was a cross-sectional observational study conducted via an online survey.

### Ethics

The study protocol was approved by the Ethics Committee of the Catholic University of Croatia, issued on April 24, 2020 (approval number: Klasa 641–03/20–01/07; Urbroj: 498–03–02-06-02/1–20-02). All participants gave written consent to participate before starting the survey.

### Setting

The study was conducted in 9 higher education institutions in Croatia that offer university-level programs in health sciences, located in cities of Dubrovnik, Osijek, Pula, Rijeka, Split, Varaždin, Zadar and Zagreb. All these institutions provided face-to-face education for health sciences students before the pandemic. Based on the decision of the Croatian governments, all institutions switched to e-learning only starting with March 16, 2020. None of the institutions provided exclusive e-learning before, but all institutions had access to the internet and online tools, including Microsoft Teams, and other similar tools provided by the Croatian government. All institutions have had internet access for years, provided by the Croatian Academic and Research Network (CARNet). All institutions had information technologies office, which was able to assist students and teachers with digital technologies. The study was conducted from April 28, 2020, to May 11, 2020.

### Participants

The participants were health sciences students of nursing, obstetrical nursing, physical therapy, radiologic technology, and diagnostic laboratory medicine studying at participating higher education institutions in Croatia in baccalaureate and master’s programs in academic year 2019/2020, who were attending courses at the time of the survey administration. There were no age or sex restrictions for participation. Students that were enrolled but have completed their courses in the previous academic year(s) and did not have an obligation to attend courses were excluded.

### Survey

We developed a new questionnaire for this study (Additional file 1). The team included in the development of a questionnaire included 8 authors with expertise in medical education, pedagogy, psychology, and research methodology; this team also included a student representative attending a university nursing program.

The questionnaire has 13 thematic sections, including general satisfaction; personal experience and engagement with e-learning; motivation and attendance; possibility to participate in e-learning; efforts invested by the institution; structure, implementation and organization of e-learning; employment and e-learning; concerns regarding lack of practical education; concerns regarding the final/diploma thesis; continuation of education during the pandemic; future of theoretical education; open-ended questions; demographics and personal characteristics. The questionnaire had a total of 73 items.

Participants respond to items using a 5-point Likert scale, closed responses, and open questions where participants can write freely their responses. The questionnaire collected information about the following aspects regarding the complete switch to e-learning: students’ satisfaction with e-learning overall, personal adjustment to e-learning, student perception of institutional adjustment to e-learning, attitudes, and concerns of students regarding acquiring knowledge, lack of practical education, completion of their theses, completion of their studies and future employment. The questionnaire also collected demographic information including age, marital status, having children, year of studies, study program (baccalaureate or master’s), the field of studies (health sciences profession), work status, full time, or part-time studying, and institution that a student attends. Information about the sex of participants was withheld because of the predominance of women among the health sciences students, due to concerns that information about sex would compromise the anonymity of men participants.

The survey was created in Google Forms. We piloted the survey on a sample of 25 students attending medical and dental university courses; we did not invite health sciences students for piloting so that we do not need to exclude them from the main study; medical and dental students that participated in piloting belong to the same age demographics, and have also had a complete switch to exclusive e-learning. Feedback of students who participated in the pilot testing was used to revise the survey. We analyzed data only from fully completed and submitted surveys.

### Survey distribution

Study authors from participating institutions contacted their students via e-mail and invited them to take part in the study. After the initial email, three more reminders were sent to students, spaced 4 days apart. We reported the total response rate, and response rate after each reminder.

### Sample size

We invited all eligible students attending participating institutions to take part in the study.

### Analysis of open text field

The questionnaire included four open-ended questions, which allowed participants to answer in open text format. We used thematic analysis to identify, analyze, and interpret recurring patterns of meaning, i.e. themes, within the qualitative responses. Each response was categorized by one author, and then a second author reviewed all categorizations to ensure uniformity.

### Data analysis

We presented numerical data as frequencies and percentages. In the text, responses “completely disagree” and “disagree” were reported in the manuscript as disagreement with the rated statements, and responses “completely agree” and “agree” as agreement; frequency of responses to all items on Likert scales were reported in detail in the tables. We did not calculate Cronbach’s alpha for this questionnaire because each item represented a unique context rather than an underlying latent construct. We explored associations of students’ characteristics with responses using a chi-square test. For analyses, we used Microsoft Excel (Microsoft Inc., Redmond, WA, USA).

## Results

### Response rate

From the 3582 eligible participants, we received 2520 (70.3%) completed surveys. After the initial invitation, we had 1701 (47.5%) responses, followed by 2050 (57.2%) after the first reminder, 2334 (65.0%) after the second reminder, and a total of 2520 (70.3%) responses after the third reminder. Response rate in individual institutions ranged from 62.0 to 82.0% (Additional file 2: Table S1). 

### Characteristics of participants

The mean age of participants was 25.7 ± 7.7 years. Most of the participants were in a relationship or single; 19.6% had children. Students were mostly at the baccalaureate level; the majority were not employed (Table 1).

**Table 1:** Characteristics of participants (*N* = 2520)

Variable	Result
Age, M ± SD	25.7 ± 7.7 years
Marital status, N (%)	
Married	501 (19.9)
In a relationship	1101 (43.7)
Single	918 (36.4)
Do you have any children? N (%)	
Yes	493 (19.6)
No	2027 (80.4)
Study level and year, N (%)	
Baccalaureate 1st year	730 (29.0)
Baccalaureate 2nd year	598 (24.0)
Baccalaureate 3rd year	603 (24.0)
Master’s 1st year	357 (14.0)
Master’s 2nd year	232 (9.0)
Higher education institution, N (%)	
Faculty of Dental Medicine and Health, Osijek	679 (26.9)
Department of Nursing, University North	502 (19.9)
Juraj Dobrila University of Pula, Medical School	342 (13.6)
Catholic University of Croatia	265 (10.5)
University Department of Health Studies, University of Split	236 (9.4)
Libertas International University	169 (6.7)
Department of Health Studies, University of Zadar	140 (5.6)
Faculty of Health Studies, University of Rijeka	121 (4.8)
University of Dubrovnik, Department of Nursing	66 (2.6)
Currently employed, N (%)	
Yes	1119 (55.6)
No	1401 (44.4)
Do you have permanent employment? N (%)	
Yes	1001 (39.7)
No	1519 (60.3)

Acronyms: *M* Mean, *SD* Standard deviation

### Experiences and engagement with e-learning

The average participants’ satisfaction with e-learning was 3.7 ± 1.1 out of maximum 5. Students’ satisfaction scores ranged from 3.00 ± 1.09 to 4.03 ± 0.9 in individual institutions (Additional file 3: Table S2). Compared with classic learning mode, about a third of participants had a neutral opinion, 39.6% found e-learning better and 24.9% found e-learning worse (detailed responses are shown in Additional file 3: Table S2).

The majority of participants were satisfied with how fast they have adjusted to e-learning (72.3%) (Table 2). There were 44.6% of students who indicated that they participate in e-courses with questions and comments just like during regular classes. About half of students indicated that they miss classroom lessons (47.5%) and in-person communication with teachers (52.5%). Just over half of the students agreed that e-learning cannot compensate for practical education and seminars (51.7%), while more than half (51.7%) disagreed with the statement that the e-learning is a complete waste of time for health sciences students (Table 2).

**Table 2:** Participants’ personal experience and engagement with exclusive e-learning (*N* = 2520)

Item	Completely disagree, N (%)	Disagree, N (%)	Neither agree nor disagree, N (%)	Agree, N (%)	Completely agree, N (%)
I am satisfied with how fast I have adjusted to e-learning	122 (4.8)	195 (7.7)	382 (15.2)	869 (34.5)	952 (37.8)
I participate in the course with questions and comments, just like during regular classes	279 (11.0)	435 (17.3)	682 (27.1)	670 (26.6)	454 (18.0)
I miss classroom lessons	361 (14.3)	398 (15.8)	565 (22.4)	511 (20.3)	685 (27.2)
I miss in-person communication with teachers	261 (10.4)	311 (12.4)	623 (24.7)	611 (24.2)	714 (28.3)
E-learning is a complete waste of time for health sciences students	822 (32.6)	630 (25.0)	645 (25.6)	253 (10.0)	170 (6.8)
E-learning cannot compensate for practical education and seminars	344 (13.7)	328 (13.0)	546 (21.6)	519 (20.6)	783 (31.1)

Compared to classroom lessons, 48.5% of the participants indicated that they are equally motivated to participate in e-learning, 55.7% indicated that they attend e-learning equally often; 51.1% of participants expressed equal motivation to participate in such lessons the longer the e-learning continued; 43.4% indicated that they felt equally connected with their colleagues and teachers, and 40.8% indicated that e-learning required equal time compared to classic classroom lessons (Additional file 4: Table S3).

The majority of students indicated that they have sufficient information technology skills to participate in e-learning independently (84.7%), that they have an adequate internet connection at home (83.7%), that they have a computer at home that they can use for e-learning without interruption (86.0%) and that they have other equipment at home, besides the computer, that enables them to participate in e-learning (65.8%) (Additional file 5: Table S4).

### Perception of efforts invested by a higher education institution towards exclusive e-learning

The majority of participants agreed that their higher education institution adapted quickly to e-learning (68.9%), organized e-learning adequately (68.7%), provided students with training about the teaching tools and software used for e-learning (59.7%), provided timely information regarding the provision of e-learning (65.8%), and that information technology office or another service was at their disposal for solving possible technical problems related to e-learning (52.0%). When asked whether their institution had expressed willingness to help students in the provision of equipment needed for participation in e-learning, 43.6% of students disagreed, while 24.7% agreed (Table 3).

**Table 3:** Efforts invested by the higher education institution in order to enable students to participate in e-learning

Item	Completely disagree, N (%)	Disagree, N (%)	Neither agree nor disagree, N (%)	Agree, N (%)	Completely agree, N (%)
My institution quickly adapted to e-learning	104 (4.1)	192 (7.6)	489 (19.4)	872 (34.6)	863 (34.3)
My institution has organized e-learning adequately	113 (4.4)	220 (8.7)	458 (18.2)	843 (33.5)	886 (35.2)
My institution has provided students with training about the teaching tools and software used for e-learning	251 (10.0)	345 (13.7)	610 (24.2)	697 (27.6)	617 (24.5)
My institution is providing timely information regarding the provision of e-learning	117 (4.6)	247 (9.8)	498 (19.8)	850 (33.7)	808 (32.1)
For solving possible technical problems related to e-learning, an information technologies office or another service is at our disposal	194 (7.7)	298 (11.8)	719 (28.5)	690 (27.4)	619 (24.6)
My institution has expressed willingness to help students in provision of equipment needed for participation in e-learning	649 (25.8)	449 (17.8)	800 (31.7)	296 (11.7)	326 (13.0)

### Perception about the structure, implementation, and organization of e-learning

Most of the students agreed with the following statements about the majority of teachers: they receive timely feedback (69.5%), the teachers’ instructions were tailored to e-learning (68.4%), teachers made effort to enable students to follow e-learning more easily (66.9%), teachers verified whether students understood the lessons by asking feedback (70.4%), the tasks and activities provided during lessons and homework usually helped students to understand the course material better (55.3%). Most of the participants agreed that teachers have generally organized themselves and adapted to e-learning well (63.4%), and most of them used e-learning software chosen by the institution (74.1%). The majority of students agreed that, generally, teaching materials were adequate for the technical demands of e-learning (67.2%) (Table 4).

**Table 4:** Structure, implementation and organization of e-learning

Item	Completely disagree, N (%)	Disagree, N (%)	Neither agree nor disagree, N (%)	Agree, N (%)	Completely agree, N (%)
I receive timely feedback from the majority of teachers	98 (3.9)	202 (8.0)	467 (18.5)	1044 (41.4)	709 (28.1)
The instructions given by the majority of teachers (e.g., about participation in lessons, modes of examination, solving tasks, or writing a seminar) are tailored to e-learning	96 (3.8)	203 (8.1)	496 (19.7)	936 (37.1)	789 (31.3)
Most of the teachers are making an effort to enable me to follow e-learning more easily, for example, by highlighting the key elements of the lecture or highlighting the transition to new content	122 (4.9)	212 (8.4)	499 (19.8)	936 (37.1)	751 (29.8)
The majority of teachers verifies whether we have understood the lessons by seeking feedback or encouraging us to ask questions	129 (5.1)	191 (7.6)	425 (16.9)	932 (37.0)	843 (33.4)
The tasks and activities that teachers provide during lessons or for homework usually help me to understand the course material better	177 (7.0)	273 (10.8)	678 (26.9)	829 (33.0)	563 (22.3)
Generally, the teaching materials are adequate for the technical demands of e-learning	112 (4.4)	203 (8.0)	513 (20.4)	970 (38.5)	722 (28.7)
The majority of teachers provides video-conferences (video-lessons)	384 (15.2)	313 (12.4)	359 (14.3)	665 (26.4)	799 (31.7)
Most of the teachers hold classes according to the official schedule	195 (7.7)	204 (8.1)	352 (14.0)	835 (33.1)	934 (37.1)
Most of the teachers are following the official curriculum	111 (4.4)	140 (5.5)	426 (17.0)	927 (36.8)	916 (36.3)
Some teachers mostly do not hold online lectures, but send students a presentation instead	683 (27.1)	461 (18.3)	407 (16.1)	427 (16.9)	542 (21.6)
Most of the teachers of classes use software that the institution chose for e-learning	82 (3.3)	116 (4.6)	452 (17.9)	852 (33.8)	1018 (40.4)
I feel left to my own devices during e-learning	409 (16.2)	389 (15.4)	708 (28.1)	493 (19.6)	521 (20.7)
Teachers have generally organized themselves and adapted to e-learning well	144 (5.7)	211 (8.4)	571 (22.7)	866 (34.4)	728 (29.0)
My expectations related to e-learning in these circumstances have been fulfilled	203 (8.1)	265 (10.5)	513 (20.4)	809 (32.1)	730 (28.9)
I am satisfied with how fast adjustment to e-learning occurred	142 (5.6)	186 (7.4)	446 (17.8)	878 (34.8)	868 (34.4)

Regarding the teaching modes, based on participants’ responses, the majority of teachers provided video-conferences (video-lessons) (55.1%), held classes according to the schedule (70.2%), and followed the official curriculum (73.1%). When asked about whether some teachers only send them presentations, instead of holding online lectures, 38.5% agreed. A third of the student indicated they felt left to their own devices during e-learning (Table 4).

Expectations related to e-learning, under the circumstances, were fulfilled for the majority of students (61.0%), and the majority indicated they were satisfied with how fast adjustment to the e-learning occurred (69.2%) (Table 4).

### Employment and e-learning

More than half of students (55.6%) indicated being currently employed. Only those students got questions about combining employment and e-learning. Among those students, few indicated that due to employment they cannot participate in e-learning (9.6%). The majority indicated that due to employment sometimes they are unable to participate in e-learning (59.7%). There were no predominant answers regarding difficulties concentrating on e-learning because of the nature of their employment (Table 5). The majority of students disagreed with the statement they could participate in e-learning at work (59.0%). Most of the students agreed that e-learning could be a good complement to classic classroom learning once the COVID-19 pandemic is over (63.8%), and that e-learning should be continued for part-time students, even after the COVID-19 pandemic is over (65.2%) (Table 5).

**Table 5:** Participants’ employment and e-learning (*N* = 1119)

Item	Completely disagree, N (%)	Disagree, N (%)	Neither agree nor disagree, N (%)	Agree, N (%)	Completely agree, N (%)
Because of my employment, I cannot participate in e-learning	215 (19.2)	359 (32.1)	438 (39.1)	70 (6.3)	37 (3.3)
Because of my employment, I am sometimes unable to participate in e-learning	108 (9.6)	132 (11.8)	211 (18.9)	444 (39.7)	224 (20.0)
Because of the nature of my employment, I find it hard to concentrate on e-learning	165 (14.7)	207 (18.6)	357 (31.9)	263 (23.5)	127 (11.3)
I have working conditions that allow me to participate in e-learning during my working hours	409 (36.6)	251 (22.4)	206 (18.4)	151 (13.5)	102 (9.1)
E-learning could be good complement to classic classroom learning once the COVID-19 pandemic is over	104 (9.3)	82 (7.3)	219 (19.6)	311 (27.8)	403 (36.0)
E-learning should be continued for part-time students, even after the COVID-19 pandemic is over	104 (9.3)	76 (6.8)	209 (18.7)	218 (19.5)	512 (45.7)

### Concerns regarding the lack of practical education

Although there were no predominant opinions regarding being deprived or concerned because of the lack of practical education (Table 6), almost half of the students (47.4%) agreed that they were afraid that it will not be possible to compensate for the lack of practical education during their studies, and the majority (55.1%) indicated that they were afraid that the lack of practical education will have permanent consequences in terms of their future job preparedness (Table 6).

**Table 6:** Participants’ concerns regarding the lack of practical education during e-learning due to the COVID-19 pandemic

Item	Completely disagree, N (%)	Disagree, N (%)	Neither agree nor disagree, N (%)	Agree, N (%)	Completely agree, N (%)
I feel deprived because of the lack of practical education	416 (16.5)	472 (18.7)	656 (26.0)	491 (19.5)	485 (19.3)
I am concerned about the lack of practical education	389 (15.4)	475 (18.9)	575 (22.8)	536 (21.3)	545 (21.6)
I am afraid that it will not be possible to compensate for the lack of practical education during my studies	520 (20.6)	674 (26.8)	593 (23.5)	363 (14.4)	370 (14.7)
I am afraid that the lack of practical education will have permanent consequences in terms of my future job preparedness	701 (27.8)	687 (27.3)	564 (22.4)	288 (11.4)	280 (11.1)

### Concerns regarding the completion of the final/diploma thesis

There were 780 (31.0%) students who indicated that they had already defined the topic of their final/diploma thesis. Specific questions shown only to those students indicated that 36.4% of the students were concerned that due to the current pandemic they will not be able to finish the work needed for finalizing their final/diploma thesis. The majority (55.5%) indicated that they were afraid that due to the current pandemic they will not be able to complete their final/diploma thesis within the planned time. There were 42.1% of students concerned that they may not be able to complete the current academic year due to problems with implementing their final/diploma thesis. Half (50.2%) indicated they were not afraid for their future employment due to potential problems with the final/diploma thesis, while 31.2% expressed they were afraid in that respect (Table 7).

**Table 7:** Concerns regarding the planned work on the final/diploma thesis (*N* = 780)

Item	Completely disagree, N (%)	Disagree, N (%)	Neither agree nor disagree, N (%)	Agree, N (%)	Completely agree, N (%)
I am afraid that due to the current pandemic I will not be able to finish the work needed for finalizing my final/diploma thesis	140 (18.0)	185 (23.7)	171 (21.9)	153 (19.6)	131 (16.8)
I am afraid that due to the current pandemic I will not be able to complete my final/diploma thesis within the planned time	103 (13.2)	130 (16.7)	114 (14.6)	221 (28.3)	212 (27.2)
I am afraid that due to problems with implementing my final/diploma thesis, I will not be able to complete it in the current academic year	142 (18.2)	175 (22.4)	135 (17.3)	168 (21.6)	160 (20.5)
I am afraid that due to problems with implementing my final/diploma thesis, I will not be able to find employment when I planned to do so	219 (28.0)	173 (22.2)	145 (18.6)	110 (14.1)	133 (17.1)

### Continuation of education during the pandemic

Most (53.9%) of the students disagreed that practical education still needs to be organized during the pandemic; 37.1% agreed that students should have suitable practical roles in health care, so they can help resolve the current pandemic; 37.5% agreed that students preparing final/diploma thesis should immediately make alternative plans that can be completed under the current circumstances; 45.2% of students agreed that e-learning needs to be improved (Table 8).

**Table 8:** Continuation of education during the pandemic (*N* = 2520)

Item	Completely disagree, N (%)	Disagree, N (%)	Neither agree nor disagree, N (%)	Agree, N (%)	Completely agree, N (%)
Despite the pandemic, practical education needs to be organized for students	739 (29.3)	620 (24.6)	600 (23.8)	302 (12.0)	259 (10.3)
Students should have suitable practical roles in health care, so they can help resolve the current pandemic	368 (14.6)	420 (16.7)	797 (31.6)	630 (25.0)	305 (12.1)
Students preparing final/diploma thesis should immediately make alternative plans that can be completed under the current circumstances	217 (8.6)	309 (12.3)	1048 (41.6)	584 (23.2)	362 (14.3)
E-learning needs to be improved	177 (7.0)	357 (14.2)	846 (33.6)	598 (23.7)	542 (21.5)

### Students’ suggestions

Having the experience with e-learning, the majority of participants indicated that in the future, they would prefer to combine classic classroom and e-learning (*N* = 1403; 55.7%); 25.4% (*N* = 641) preferred to continue with classic classroom learning, and 18.9% (*N* = 476) would continue with e-learning only.

When asked to provide suggestions on how e-learning could be improved, 913 (36%) students responded. The most common suggestions/comments were to provide more online lectures instead of posting the text of presentations (*N* = 118; 13.0%), students have way too many tasks and assignments (*N* = 48; 5.3%), teachers need to be engaged more (*N* = 44; 4.8%), online exams need to be introduced (*N* = 35; 3.8%), lectures should be video-taped and videos provided to students (*N* = 27; 2.9%), better communication and availability of professors (*N* = 26; 2.8%), pdf materials for independent learning need to be provided (*N* = 25; 2.7%), teachers need to be educated about using technology (*N* = 24; 2.5%), More details are given in Additional file 6: Table S5.

There were 886 (35.0%) responses to the question about suggestions for compensating the students for their lack of practical education. The most common suggestions were: providing video-materials/tutorials (*N* = 80; 9.0%), for students who already work as nurses such compensation is not necessary (*N* = 64; 7.2%), compensating when the circumstances will allow (*N* = 62; 7.0%), compensating with assignments and case studies (*N* = 50; 5.6%), compensating in the next academic year (*N* = 44; 5.0%), and teaching in smaller groups of students (*N* = 39; 4.4%). More details are given in Additional file 7: Table S6.

Question about providing suggestions for students who could face problems with completing their final/diploma thesis under the circumstances was responded by 620 (25.0%) students. The most common suggestions were to extend deadlines (*N* = 65; 10.4%), enable online thesis defense (*N* = 50; 8.0%), change or adjustment of a thesis topic (*N* = 39; 6.3%), ensuring the availability of a mentor (*N* = 38; 6.1%), students should conduct research for their theses via online questionnaires (*N* = 38; 6.1%), ensuring cooperativeness of mentors (*N* = 22; 3.5%). More details are given in Additional file 8: Table S7.

Students were also invited to leave any comment that they might have regarding the potential permanent consequences of e-learning, i.e. lack of practical education, on their education and professional development; 637 (25%) students responded. The majority of students indicated that there will be no major/permanent consequences of the lack of practical education (*N* = 112; 18.0%). It was also indicated that students who already work as nurses (*N* = 39; 4.1%) or who have completed nursing high school (*N* = 27; 4.2%) will have fewer consequences from the lack of practical education compared to other students. More details are given in Additional file 9: Table S8.

### Differences in responses based on participants’ characteristics

In Additional file 10 we have provided analysis of each response, based on different participant characteristics. For each response, there is descriptive statistics reported and differences between the subgroups (Additional file 10). All raw data collected within the study are reported in Additional file 10, together with the results of the chi-square test.

## Discussion

The main finding of our study is that the majority of university-level health sciences students in Croatia have adjusted well to the exclusive e-learning during the COVID-19 pandemic and the majority considered that their institutions adjusted well too.

COVID-19 became a global public health issue very quickly; the World Health Organization (WHO) was informed about the emerging threat in China on December 31, 2019 and pandemic was declared on March 11, 2020 [13]. Many governments around the world responded with lockdowns, cessation of classroom education, and the complete switch to distance learning. However, not all types of education can be successfully implemented via e-learning. This is particularly problematic for all aspects of practical education where experiences are crucial, and skills need to be learned. Medical and health sciences students need contacts with patients to learn the necessary skills and to be personally exposed to patient care. Thus, warnings have been voiced about potential consequences of the COVID-19 pandemic for medical education, warning that medical students may be heavily deprived due to the lack of practical education, and proposing that medical students could even help during the pandemic [14]. The same arguments can be used for health sciences education. However, despite the explosion of research about COVID-19 [15], few studies have been devoted to the exploration of radical changes that the pandemic imposed in medical and health sciences education.

Compton et al. surveyed medical students of Duke-NUS Medical School in Singapore, less than a month after the pandemic onset. They found that about a third of the 179 surveyed students would not prefer to return to the clinical setting; the students’ willingness to return to the clinical environment was associated with personal characteristics and students’ perceptions about the risk to the healthcare systems [16, 17].

Kumar Srinivasan described experiences of medical students and an anatomy teacher regarding the use of an e-learning platform for anatomy tutorials on the Zoom platform during the COVID-19 pandemic in Singapore. The report describes the general satisfaction of students, as well as students’ feedback about how the lessons and tutorials could be improved [6]. Pather et al. conducted a qualitative study to document the experiences of anatomy teachers in New Zealand regarding changes in anatomy education during the COVID-19 pandemic. They highlighted that the teachers have embraced the change. However, teachers were also concerned about the lack of student-teacher interaction, student performance, and satisfaction. It is also highlighted that bridges made during the pandemic could impact the future of anatomy education [18].

One such potential “bridge” could be incorporating e-learning into “classic” classroom-based curricula. Most of the students in our study indicated that in the future they would appreciate more a combination of e-learning and classroom learning, compared to either of those alone. This could be in line with the findings of a study suggesting that the effect of e-learning in nursing education is situational when compared to conventional learning [19].

While most students in our study agreed that they and their institutions/teachers had adapted quickly to the exclusive e-learning, that they had adequate information technologies at home to participate in the e-learning, and that institutions supported them in technological adjustment, this was not unanimous. This indicates that some students and institutions may have needed more time to adjust to exclusive e-learning.

Participants’ responses indicated that most teachers were engaging with students via video-lectures, on time, and in line with the curriculum. However, it appears that some teachers send students only presentations, instead of lecturing online. Indeed, one of the most frequent suggestions regarding improvement of e-learning experience was students’ wish that teachers should not only post presentations online for students to study when they want; instead, students wanted video-lectures with teachers. Others suggested that videotaped lectures should be made available to students so that students can watch and rewind them at their convenience.

Disruptions caused by the pandemic may prevent some students from completing their research studies planned for their final/diploma theses. Thus, it was important to the hear opinions of students about what could be done for students facing this potential problem. Most students suggested extending deadlines. For example, students are usually expected to complete and defend their thesis within the academic year in which they enrolled their final year of the study program. Otherwise, they need to enroll another year of studies and pay tuition for this additional study year. Based on students’ feedback, participating institutions are now extending those deadlines, enabling students who have faced obstacles in completing their research during the pandemic to do it over a longer period.

It is commendable that some institutions have offered students help with the technology needed for participating in e-learning. Our study did not address the financial cost of e-learning for students, but it needs to be emphasized that it should not be presumed that all students have all equipment at home that they can use exclusively. A large part of our student body were students studying part-time, which are generally older than students who enroll in health sciences programs immediately after high schools. Part-time students are usually health sciences professionals who have completed health sciences high schools in Croatia, and who have been working for a while in health care institutions, now wishing to attain higher education due to adjustment of Croatian professional curricula with EU regulations. Thus, if students have a family and children, and everyone is expected to work and study at home during the pandemic, the family may not have had, for example, a laptop for each child and each parent at home. Judging by the students’ responses, there were indeed those that did not have all the necessary equipment at home for proper participation in e-learning.

Many students did not express concern due to a lack of practical lessons, voicing opinions that this can be compensated once they start working. This could indicate that students are underestimating the importance of practical education during their university studies.

Lessons learned in this study are multiple. COVID-19 has presented a powerful test of the potential of online learning; it appears that students and institutions have passed the test. Adult university students, with wide age ranges, have on average adjusted well to exclusive e-learning and embraced the online environments. Students predominantly judged that their higher education institutions have also adjusted well to the abrupt switch to e-learning. Many students would prefer to continue studying online or combining face-to-face and e-learning. Institutions should interact with their students via online surveys to obtain feedback that will enable them to improve on their delivery of online education and organizational matters. Our study showed that students had many suggestions, from asking different technological platforms, different modes of teaching materials and delivery of lessons, extending deadlines for theses, and changing mode of conducting research for their theses. Feedback provided by students can be used to adjust online teaching to learner realities; for example, students suggested that lectures should be videotaped and posted for later viewing. Many students in our sample were employed, and technology could thus help them make their e-learning environment more flexible. A combination of recorded and live stream teaching could help in enabling student-centered learning. Digital literacy skills are important aspects now required from both students and teachers; it should be incorporated into the university curriculum and continuing education of teachers. Our results emphasize the importance of infrastructure (such as networks and devices), educational platforms (in terms of their stability, interactions that they enable, options they provide, ability to adapt), and preparedness of students and teachers. An important aspect of educating health sciences students is practical education; learning how to care for a patient. While most students in our study did not express much concern regarding a lack of practical education, as most of them expected that they will learn on the job, and many were already employed in healthcare, this is a crucial part of their education that is difficult to replace with online content. Thus, blended learning in health sciences, combining face-to-face and e-learning, where practical education will be delivered in person, might become the new norm, even after the COVID-19 pandemic.

### Limitations

One of the study limitations is that we created a completely new questionnaire for this study. We were unable to find a similar questionnaire in the literature, about exclusive e-learning for health sciences students, or medical students, that we could have adapted to our needs. For transparency, we have reported our manuscript in full in a supplementary file, so that it can be reused or modified.

Secondly, we did not collect data on the sex of participants. However, in university health sciences studies in Croatia there is an overwhelming predominance of women. If we were to collect data on sex, together with all the other personal characteristics that we were collecting, we were concerned that men would be easily identified, and thus by avoiding this question we helped preserve participants’ anonymity.

Thirdly, our questions were phrased to collect the opinion of students regarding “the majority” of teachers and courses. We acknowledge that they may be differences between different teachers and courses that may impact the individual experiences, but in this study, we were interested in their opinions and attitudes towards the overall experience of exclusive e-learning. Likewise, there may be inherent differences between the institutions that may have influenced the results, as some schools had more students compared to others. For example, Libertas International University has created online simulations for students that could be used for exercising practical skills. However, the aim of this study was not to focus on individual results from each school but to analyze overall collective students’ opinion. Each school that participated in this study will analyze separately responses of their own students, to consider students’ feedback and suggestions and use it towards the improvement of e-learning experience.

In our study, we have included only students and not teachers. Perspectives of teachers regarding the complete switch to online learning would be valuable and should be explored in future studies.

### Strengths

The main strength of this study is the inclusion of 9 out of 10 higher education institutions offering university-level health sciences education in Croatia. Furthermore, the response rate of students was above 70%. Thus, our results should be considered representative of the opinions and attitudes of health sciences students in Croatia.

At the time of writing the last version of this manuscript (September 2020), the epidemiological situation in Croatia regarding COVID-19 was worsening [2]. Due to the unpredictable situation, it was unclear whether the exclusive e-learning will continue in the new academic year. Faced with potential continued online learning, institutions can use students’ feedback obtained in this study to improve e-learning and enhance the student experience.

## Conclusion

Most university-level health sciences students in Croatia were satisfied with the exclusive e-learning during the COVID-19 pandemic in early 2020, as well as their personal and institutional adjustment to the new normal in university education. Exploring students’ satisfaction and towards e-learning and seeking feedback about measures for improvement can help institutions to improve the exclusive e-learning experience for students in the time of the pandemic.

## Supplementary Information

**Additional file 1.** Survey used in the study.

**Additional file 2: Table S1.** Response rate in each institution.

**Additional file 3: Table S2.** Rating the e-learning experience.

**Additional file 4: Table S3.** Motivation and connection with fellow colleagues and teachers (*N* = 2520).

**Additional file 5: Table S4.** Possibility of students’ participation in e-learning, based on their information technologies skills and availability of equipment at home (*N* = 2520).

**Additional file 6: Table S5.** Students’ suggestions/comments on how e-learning could be improved (*N* = 920). Responses given by more than 10 students are shown in detail.

**Additional file 7: Table S6.** Students’ suggestions/comments for compensating the students for their lack of practical education (*N* = 886). Responses given by more than 10 students are shown in detail.

**Additional file 8: Table S7.** Students’ suggestions/comments for students who may have problems with completing their final/diploma thesis due to pandemic (*N* = 620). Responses given by more than 10 students are shown in detail.

**Additional file 9: Table S8.** Students’ suggestions/comments regarding the potential permanent consequences of e-learning, i.e. lack of practical education, on their education and professional development (*N* = 637). Responses given by more than 5 students are shown in detail.

**Additional file 10.** Raw data collected within the study and results of the chi-square test.

## Data Availability

Raw data collected within this study are included in the Additional file 10, which accompanies the manuscript.

## Abbreviation

CARNet: Croatian Academic and Research Network
