# Supplementary material for: Attitudes and concerns of undergraduate university health sciences students in Croatia regarding complete switch to e-learning during COVID-19 pandemic: a survey
Source: BMC Med Educ. 2020 Nov 10;20:416. doi: 10.1186/s12909-020-02343-7 (PMC7652670; doi:10.1186/s12909-020-02343-7)
Supplement: Supplementary file 1 — Additional file 1. Survey used in the study. [file 12909_2020_2343_MOESM1_ESM.docx]

**Supplementary file 1. Survey about exclusive e-learning for health sciences students during the COVID-19 pandemic**

Dear Colleagues,

We have been forced to implement exclusive e-learning during the COVID-19 for more than a month now, and it is uncertain how long you will need to attend courses in this way. Therefore, we have decided to conduct a survey about the satisfaction and attitudes of health sciences students toward exclusive e-learning and its potential consequences. It takes no more than 15 minutes to complete the survey. Participation in the survey is anonymous and voluntary. Your participation in the survey will contribute to the improvement of courses and help the institution adjust to the new circumstances.

Please rate your general satisfaction with the overall e-learning that has been provided thus far:

| 1 – completely dissatisfied | 2 - dissatisfied | 3 – neither satisfied nor dissatisfied | 4 - satisfied | 5 – completely satisfied |
| --- | --- | --- | --- | --- |

**How would you rate the e-learning you have had so far, compared to the classic classroom learning you had before?**

e-learning is:

| 1 – much worse | 2 - worse | 3 – neither better nor worse | 4 - better | 5 – much better |
| --- | --- | --- | --- | --- |

**Please rate these statements regarding your experience and engagement with e-learning:**

| Item | 1 – completely disagree | 2 - disagree | 3 – neither agree nor disagree | 4 – agree | 5 – completely agree |
| --- | --- | --- | --- | --- | --- |
| I am satisfied with how fast I have adjusted to e-learning |  |  |  |  |  |
| I participate in the course with questions and comments, just like during regular classes |  |  |  |  |  |
| I miss classroom lessons |  |  |  |  |  |
| I miss in-person communication with teachers |  |  |  |  |  |
| E-learning is a complete waste of time for health sciences students |  |  |  |  |  |
| E-learning cannot compensate for practical education and seminars |  |  |  |  |  |

Compared to classroom lessons, I am motivated to participate in e-learning:

More, Equally, Less

Compared to classroom lessons, I attend e-learning:

Less frequently, Equally, More frequently

The longer the e-learning continues, my motivation to participate in such lessons:

Increases, Remains equal, Decreases

Compared to classroom learning, during e-learning I am connected with my colleagues and teachers:

More, Equally, Less

Regarding time, compared to classic classroom lessons, e-learning requires:

More time, Equal time, Less time

**Please rate your level of agreement with the following statements, related to the possibility of your participation in e-learning, based on your information technologies skills and availability of equipment at home:**

| Item | 1 – completely disagree | 2 - disagree | 3 – neither agree nor disagree | 4 – agree | 5 – completely agree |
| --- | --- | --- | --- | --- | --- |
| I have sufficient information technology skills to participate in e-learning independently |  |  |  |  |  |
| I have Internet at home, which enables me to participate in e-learning without interruption |  |  |  |  |  |
| I have a computer at home that I can use without interruption for e-learning |  |  |  |  |  |
| I have other equipment at home, besides a computer, that enables me to participate in e-learning |  |  |  |  |  |

**Please state your agreement with the following statements related to the efforts invested by your higher education institution in order to enable you to participate in e-learning**

| Item | 1 – completely disagree | 2 - disagree | 3 – neither agree nor disagree | 4 – agree | 5 – completely agree |
| --- | --- | --- | --- | --- | --- |
| My institution quickly adapted to e-learning |  |  |  |  |  |
| My institution has organized e-learning adequately |  |  |  |  |  |
| My institution has provided students with training about the teaching tools and software used for e-learning |  |  |  |  |  |
| My institution is providing timely information regarding the provision of e-learning |  |  |  |  |  |
| For solving possible technical problems related to e-learning, an information technologies office or another service is at our disposal |  |  |  |  |  |
| My institution has expressed willingness to help students in provision of equipment needed for participation in e-learning |  |  |  |  |  |

**Please rate your level of agreement with the following statements related to the structure, implementation and organization of e-learning**

| Item | 1 – completely disagree | 2 - disagree | 3 – neither agree nor disagree | 4 – agree | 5 – completely agree |
| --- | --- | --- | --- | --- | --- |
| I receive timely feedback from the majority of teachers |  |  |  |  |  |
| The instructions given by the majority of teachers (e.g., about participation in lessons, modes of examination, solving tasks, or writing a seminar) are tailored to e-learning |  |  |  |  |  |
| Most of the teachers are making an effort to enable me to follow e-learning more easily, for example, by highlighting the key elements of the lecture or highlighting the transition to new content |  |  |  |  |  |
| The majority of teachers verifies whether we have understood the lessons by seeking feedback or encouraging us to ask questions |  |  |  |  |  |
| The majority of teachers finds a way to motivate us to participate in lessons under these distance learning conditions |  |  |  |  |  |
| The tasks and activities that teachers provide during lessons or for homework usually help me to understand the course material better |  |  |  |  |  |
| Generally, the teaching materials are adequate for the technical demands of e-learning |  |  |  |  |  |
| The majority of teachers provides video-conferences (video-lessons) |  |  |  |  |  |
| Most of the teachers hold classes according to the official schedule |  |  |  |  |  |
| Most of the teachers are following the official curriculum |  |  |  |  |  |
| Some teachers mostly do not hold online lectures, but send students a presentation instead |  |  |  |  |  |
| Most of the teachers of classes use software that the institution chose for e-learning |  |  |  |  |  |
| I feel left to my own devices during e-learning |  |  |  |  |  |
| Teachers have generally organized themselves and adapted to e-learning well |  |  |  |  |  |
| My expectations related to e-learning in these circumstances have been fulfilled |  |  |  |  |  |
| I am satisfied with how fast adjustment to e-learning occurred |  |  |  |  |  |

**Are you currently employed?**

Yes

No

*The following questions are only for those participants who answered “yes”:*

**Please indicate your level of agreement with these statements related to your employment and e-learning:**

| Item | 1 – completely disagree | 2 - disagree | 3 – neither agree nor disagree | 4 – agree | 5 – completely agree |
| --- | --- | --- | --- | --- | --- |
| Because of my employment, I cannot participate in e-learning |  |  |  |  |  |
| Because of my employment, I am sometimes unable to participate in e-learning |  |  |  |  |  |
| Because of the nature of my employment, I find it hard to concentrate on e-learning |  |  |  |  |  |
| I have working conditions that allow me to participate in e-learning during my working hours |  |  |  |  |  |
| E-learning could be good complement to classic classroom learning once the COVID-19 pandemic is over |  |  |  |  |  |
| E-learning should be continued for part-time students, even after the COVID-19 pandemic is over |  |  |  |  |  |

**Questions for all the students**

**Please state your level of agreement with the following statements related to possible concerns you might have regarding the lack of practical education during e-learning due to the COVID-19 pandemic.**

| Item | 1 – completely disagree | 2 - disagree | 3 – neither agree nor disagree | 4 – agree | 5 – completely agree |
| --- | --- | --- | --- | --- | --- |
| I feel deprived because of the lack of practical education |  |  |  |  |  |
| I am concerned about the lack of practical education |  |  |  |  |  |
| I am afraid that it will not be possible to compensate for the lack of practical education during my studies |  |  |  |  |  |
| I am afraid that the lack of practical education will have permanent consequences in terms of my future job preparedness |  |  |  |  |  |

**Have you already defined the topic of your final/diploma thesis?**

Yes

No

*The following questions are only for those participants who answered “yes”:*

**Please rate your level of agreement with the following statements related to your concerns regarding the planned work on your final/diploma thesis**

| Item | 1 – completely disagree | 2 - disagree | 3 – neither agree nor disagree | 4 – agree | 5 – completely agree |
| --- | --- | --- | --- | --- | --- |
| I am afraid that due to the current pandemic I will not be able to finish the work needed for finalizing my final/diploma thesis. |  |  |  |  |  |
| I am afraid that due to the current pandemic I will not be able to complete my final/diploma thesis within the planned time |  |  |  |  |  |
| I am afraid that due to problems with implementing my final/diploma thesis, I will not be able to complete it in the current academic year |  |  |  |  |  |
| I am afraid that due to problems with implementing my final/diploma thesis, I will not be able to find employment when I planned to do so |  |  |  |  |  |

*Questions for all students*

**Please rate your level of agreement with the following statements about the continuation of your education during the pandemic**

| Item | 1 – completely disagree | 2 - disagree | 3 – neither agree nor disagree | 4 – agree | 5 – completely agree |
| --- | --- | --- | --- | --- | --- |
| Despite the pandemic, practical education needs to be organized for students |  |  |  |  |  |
| Students should have suitable practical roles in health care, so they can help resolve the current pandemic |  |  |  |  |  |
| Students preparing final/diploma thesis should immediately make alternative plans that can be completed under the current circumstances |  |  |  |  |  |
| E-learning needs to be improved |  |  |  |  |  |

Considering the experience with e-learning, what would you prefer in the future for theoretical education?

-classical classroom lessons

-e-learning

-a combination of both

Please provide your suggestions on how e-learning could be improved… (open question)

Please provide your suggestions for compensating the students for their lack of practical education … (open question)

Please provide your suggestions for students who may have problems with completing their final/diploma theses under the current circumstances … (open question)

Please provide any comments that you might have regarding the potential permanent consequences of e-learning, i.e., lack of practical education, on your education and professional development … (open question)

------

**Your age**: ___________ (years)

**Marital status:**

Married

In a relationship

Single

**Do you have any children**: yes/no

**Sex**: M/F

(note: *in our study, we opted in the end not to use this question, or question “type of study” because there are few men in the classes, and few individuals in certain “type of study” so we were very concerned that this would compromise participants’ anonymity*)

**Year of studies**: 1/2/3

**Study level:** Baccalaureate / Master’s

**Type of study:**

Nursing

Physical therapy

Midwifery

Radiological diagnostics

Medical laboratory diagnostics

(note: *in our study, we opted in the end not to use this question, or question “type of study” because there are few men in the classes, and few individuals in certain “type of study” so we were very concerned that this would compromise participants’ anonymity*)

**Your institution of higher education:**

Catholic University of Croatia

University Department of Health Studies, University of Split

Faculty of Health Studies, University of Rijeka

Juraj Dobrila University of Pula, Medical School

Libertas International University

Faculty of Dental Medicine and Health, Osijek

Department of Nursing, University North

Department of Health Studies, University of Zadar

University of Dubrovnik, Department of Nursing

**Do you have permanent employment?** Yes/No

Thank you for your participation.
