# Supplementary material for: Attitudes and concerns of undergraduate university health sciences students in Croatia regarding complete switch to e-learning during COVID-19 pandemic: a survey
Source: BMC Med Educ. 2020 Nov 10;20:416. doi: 10.1186/s12909-020-02343-7 (PMC7652670; doi:10.1186/s12909-020-02343-7)
Supplement: Supplementary file 2 — Additional file 2: Table S1. Response rate in each institution. [file 12909_2020_2343_MOESM2_ESM.docx]

# **Supplementary table 1. Response rate in each institution**

| **Higher education institution** | **n/N (%)** |
| --- | --- |
| Department of Health Studies, University of Zadar | 140/171 (81.9) |
| University of Dubrovnik, Department of Nursing | 66/84 (78.6) |
| Juraj Dobrila University of Pula, Medical School | 121/156 (77.6) |
| Department of Nursing, University North | 502/687 (73.1) |
| Faculty of Dental Medicine and Health, Osijek | 679/932 (72.9) |
| Catholic University of Croatia | 265/366 (72.4) |
| Faculty of Health Studies, University of Rijeka | 342/535 (63.9) |
| Libertas International University | 169/269 (62.8) |
| University Department of Health Studies, University of Split | 236/382 (61.8) |
