# Supplementary material for: Attitudes and concerns of undergraduate university health sciences students in Croatia regarding complete switch to e-learning during COVID-19 pandemic: a survey
Source: BMC Med Educ. 2020 Nov 10;20:416. doi: 10.1186/s12909-020-02343-7 (PMC7652670; doi:10.1186/s12909-020-02343-7)
Supplement: Supplementary file 3 — Additional file 3: Table S2. Rating the e-learning experience. [file 12909_2020_2343_MOESM3_ESM.docx]

# **Supplementary table 2. Rating the e-learning experience**

| **Item** | **Result** |
| --- | --- |
| General satisfaction of students in individual institutions, M±SD |  |
| Faculty of Dental Medicine and Health, Osijek | 4.01±0.99 |
| Department of Nursing, University North | 4.03±0.92 |
| Juraj Dobrila University of Pula, Medical School | 3.00±1.09 |
| Catholic University of Croatia | 4.02±0.93 |
| University Department of Health Studies, University of Split | 3.33±1.08 |
| Libertas International University | 3.86±1.09 |
| Department of Health Studies, University of Zadar | 3.25±1.21 |
| Faculty of Health Studies, University of Rijeka | 3.24±1.18 |
| University of Dubrovnik, Department of Nursing | 3.26±1.50 |
| Please rate your general satisfaction with the overall e-learning that was provided thus far, N (%) |  |
| Completely dissatisfied | 103 (4.1) |
| Dissatisfied | 254 (10.1) |
| Neither satisfied nor dissatisfied | 616 (24.4) |
| Satisfied | 799 (31.7) |
| Completely satisfied | 748 (29.7) |
| How would you rate e-learning you had so far, compared to the classic learning in classroom that you had before? N (%) |  |
| Much worse | 205 (8.1) |
| Worse | 424 (16.8) |
| Neither better nor worse | 893 (35.4) |
| Better | 583 (23.1) |
| Much better | 415 (16.5) |
