# Supplementary material for: Attitudes and concerns of undergraduate university health sciences students in Croatia regarding complete switch to e-learning during COVID-19 pandemic: a survey
Source: BMC Med Educ. 2020 Nov 10;20:416. doi: 10.1186/s12909-020-02343-7 (PMC7652670; doi:10.1186/s12909-020-02343-7)
Supplement: Supplementary file 4 — Additional file 4: Table S3. Motivation and connection with fellow colleagues and teachers (N = 2520). [file 12909_2020_2343_MOESM4_ESM.docx]

# **Supplementary table 3. Motivation and connection with fellow colleagues and teachers (N=2520)**

| **Item** | **N (%)** |
| --- | --- |
| Compared to classroom lessons, I am motivated to participate in e-learning: |  |
| More | 402 (16.0) |
| Equally | 1221 (48.5) |
| Less | 897 (35.6) |
| Compared to classroom lessons, I attend e-learning: |  |
| Less frequently | 372 (14.8) |
| Equally | 1403 (55.7) |
| More frequently | 745 (29.6) |
| The longer the e-learning continues, my motivation to participate in such lessons: |  |
| Increases | 363 (14.4) |
| Remains equal | 1287 (51.1) |
| Decreases | 870 (34.5) |
| Compared to classroom learning, during e-learning I am connected with my colleagues and teachers: |  |
| More | 328 (13.0) |
| Equally | 1093 (43.4) |
| Less | 1099 (43.6) |
| Regarding time, compared to classic classroom lessons, e-learning requires: |  |
| More time | 565 (22.4) |
| Equal time | 1029 (40.8) |
| Less time | 926 (36.7) |
