# Supplementary material for: Attitudes and concerns of undergraduate university health sciences students in Croatia regarding complete switch to e-learning during COVID-19 pandemic: a survey
Source: BMC Med Educ. 2020 Nov 10;20:416. doi: 10.1186/s12909-020-02343-7 (PMC7652670; doi:10.1186/s12909-020-02343-7)
Supplement: Supplementary file 5 — Additional file 5: Table S4. Possibility of students’ participation in e-learning, based on their information technologies skills and availability of equipment at home (N = 2520). [file 12909_2020_2343_MOESM5_ESM.docx]

# **Supplementary table 4. Possibility of students’ participation in e-learning, based on their information technologies skills and availability of equipment at home (N=2520)**

| **Item** | **Completely disagree, N (%)** | **Disagree, N (%)** | **Neither agree nor disagree, N (%)** | **Agree, N (%)** | **Completely agree, N (%)** |
| --- | --- | --- | --- | --- | --- |
| I have sufficient information technology skills to participate in e-learning independently | 33 (1.3) | 58 (2.3) | 294 (11.7) | 747 (29.6) | 1388 (55.1) |
| I have internet at home, which enables me to participate in e-learning without interruption | 45 (1.8) | 113 (4.5) | 250 (10.0) | 639 (25.3) | 1473 (58.4) |
| I have a computer at home that I can use without interruption for e-learning | 65 (2.6) | 121 (4.8) | 166 (6.6) | 514 (20.4) | 1654 (65.6) |
| I have other equipment at home, besides a computer, that enables me to participate in e-learning | 196 (7.7) | 254 (10.1) | 414 (16.4) | 502 (20.0) | 1154 (45.8) |
