# Supplementary material for: Attitudes and concerns of undergraduate university health sciences students in Croatia regarding complete switch to e-learning during COVID-19 pandemic: a survey
Source: BMC Med Educ. 2020 Nov 10;20:416. doi: 10.1186/s12909-020-02343-7 (PMC7652670; doi:10.1186/s12909-020-02343-7)
Supplement: Supplementary file 6 — Additional file 6: Table S5. Students’ suggestions/comments on how e-learning could be improved (N = 920). Responses given by more than 10 students are shown in detail. [file 12909_2020_2343_MOESM6_ESM.docx]

# **Supplementary table 5. Students’ suggestions/comments on how e-learning could be improved (N=920). Responses given by more than 10 students are shown in detail.**

| **Suggestion/comment** | **N (%)** |
| --- | --- |
| More online lectures instead of simply posting text of presentations | 118 (13.0) |
| Way too many tasks and assignments | 48 (5.3) |
| Teachers need to be engaged more | 44 (4.8) |
| Online exams need to be introduced | 35 (3.8) |
| Lectures should be video-taped and videos provided to students | 27 (3.0) |
| Better communication and availability of professors | 26 (2.8) |
| Pdf materials for independent learning need to be provided | 25 (2.7) |
| Teachers need to be educated about using technology | 24 (2.6) |
| Better platforms for online lessons | 21 (2.3) |
| Highlight the parts important for studying | 20 (2.2) |
| Sending notifications timely | 19 (2.1) |
| Use Zoom, Skype or something similar | 18 (2.0) |
| Shorter lectures | 18 (2.0) |
| Classic teaching is better | 18 (2.0) |
| Good internet connection is needed | 18 (2.0) |
| Having lessons and exams at the agreed time | 18 (2.0) |
| Precise instructions for solving assignments | 11 (1.2) |
| Better interaction between a lecturer and a student | 11 (1.2 |
| Better organization in providing lessons | 11 (1.2) |
| Other | 367 (40.0) |
