# Supplementary material for: Attitudes and concerns of undergraduate university health sciences students in Croatia regarding complete switch to e-learning during COVID-19 pandemic: a survey
Source: BMC Med Educ. 2020 Nov 10;20:416. doi: 10.1186/s12909-020-02343-7 (PMC7652670; doi:10.1186/s12909-020-02343-7)
Supplement: Supplementary file 7 — Additional file 7: Table S6. Students’ suggestions/comments for compensating the students for their lack of practical education (N = 886). Responses given by more than 10 students are shown in detail. [file 12909_2020_2343_MOESM7_ESM.docx]

# **Supplementary table 6. Students’ suggestions/comments for compensating the students for their lack of practical education (N=886). Responses given by more than 10 students are shown in detail.**

| **Suggestion/comment** | **N (%)** |
| --- | --- |
| Providing video-materials/tutorials | 80 (9.0) |
| For students who already work as nurses such compensation is not necessary | 64 (7.2) |
| Compensating the practical education when circumstances will allow | 62 (7.0) |
| Compensating with assignments and case studies | 50 (5.6) |
| compensating in the next academic year | 44 (5.0) |
| Teaching in smaller groups of students | 39 (4.4) |
| Compensation in a teaching cabinet | 34 (3.8) |
| Practical education needs to be done | 32 (3.6) |
| Compensate during the summer break | 29 (3.3) |
| Online practical lessons and workshops | 28 (3.2) |
| Compensation through seminars | 27 (3.0) |
| Reduce the number of hours of practical education | 27 (3.0) |
| There is no need for compensation | 26 (2.9) |
| Compensation | 24 (2.7) |
| Compensating the practical part in a place where students live | 16 (1.8) |
| Webinar, video conferences, Zoom, etc. | 15 (1.7) |
| More lectures | 13 (1.5) |
| There is no need for compensation, it will all be compensated at work | 12 (1.4) |
| Other | 264 (30.0) |
