# Supplementary material for: Attitudes and concerns of undergraduate university health sciences students in Croatia regarding complete switch to e-learning during COVID-19 pandemic: a survey
Source: BMC Med Educ. 2020 Nov 10;20:416. doi: 10.1186/s12909-020-02343-7 (PMC7652670; doi:10.1186/s12909-020-02343-7)
Supplement: Supplementary file 8 — Additional file 8: Table S7. Students’ suggestions/comments for students who may have problems with completing their final/diploma thesis due to pandemic (N = 620). Responses given by more than 10 students are shown in detail. [file 12909_2020_2343_MOESM8_ESM.docx]

# **Supplementary table 7. Students’ suggestions/comments for students who may have problems with completing their final/diploma thesis due to pandemic (N=620). Responses given by more than 10 students are shown in detail.**

| **Suggestion/comment** | **N (%)** |
| --- | --- |
| Extending deadlines | 65 (10.4) |
| Online thesis defense | 50 (8.0) |
| Change od adjustment of a thesis topic | 39 (6.3) |
| Ensuring availability of a mentor | 38 (6.1) |
| Conducting online questionnaires | 38 (6.1) |
| Cooperativeness of mentors | 22 (3.5) |
| More time | 20 (3.2) |
| Online conduct of the study and online defense | 17 (2.7) |
| Delaying deadlines for completing a thesis and defending it | 17 (2.7) |
| Online consultations | 17 (2.7) |
| Classic thesis defense in line with epidemiological measures | 13 (2.1) |
| Lower the expectations | 12 (1.9) |
| Enable easier access to the literature | 12 (1.9) |
| Other | 260 (42.0) |
