# Supplementary material for: Attitudes and concerns of undergraduate university health sciences students in Croatia regarding complete switch to e-learning during COVID-19 pandemic: a survey
Source: BMC Med Educ. 2020 Nov 10;20:416. doi: 10.1186/s12909-020-02343-7 (PMC7652670; doi:10.1186/s12909-020-02343-7)
Supplement: Supplementary file 9 — Additional file 9: Table S8. Students’ suggestions/comments regarding the potential permanent consequences of e-learning, i.e. lack of practical education, on their education and professional development (N = 637). Responses given by more than 5 students are shown in detail. [file 12909_2020_2343_MOESM9_ESM.docx]

# **Supplementary table 8. Students’ suggestions/comments regarding the potential permanent consequences of e-learning, i.e. lack of practical education, on their education and professional development (N=637). Responses given by more than 5 students are shown in detail.**

| **Suggestion/comment** | **N (%)** |
| --- | --- |
| There will be no major and/or permanent consequences | 112 (18.0) |
| Lack of practical lessons may negatively influence quality of education and professional development | 41 (6.4) |
| For students who work in nursing already, there will be no consequences | 39 (6.1) |
| Practical lessons need to be compensated | 35 (5.5) |
| Consequences will manifest as lack of necessary skills and practical knowledge | 30 (4.7) |
| Everything can be compensated | 28 (4.4) |
| There will be more consequences for students who have completed gymnasium high school, compared to those who have completed nursing high school | 27 (4.2) |
| Practical lessons cannot be replaced with any other type of education | 18 (2.8) |
| Consequences will be manifested in lagging behind or inadequate professional development | 9 (1.4) |
| Consequences will be manifested as a lack of knowledge | 9 (1.4) |
| Consequences will be manifested as a lack of motivation | 7 (1.1) |
| E-lessons are great; this type of education should remain in the future | 7 (1.1) |
| Consequences will manifest as insufficient preparedness for a future job | 5 (0.8) |
